# Food Emulsifier Glycerin Monostearate Increases Internal Exposure Levels of Six Priority Controlled Phthalate Esters and Exacerbates Their Male Reproductive Toxicities in Rats

**DOI:** 10.1371/journal.pone.0161253

**Published:** 2016-08-30

**Authors:** Hai-Tao Gao, Run Xu, Wei-Xin Cao, Xu Zhou, Ye-Hui-Mei Yan, Lingeng Lu, Qian Xu, Yang Shen

**Affiliations:** 1 Key Laboratory of Environmental Medicine Engineering, Ministry of Education, School of Public Health, Southeast University, Nanjing, 210009, China; 2 Suzhou Key Laboratory of Environment and Biosafety, Southeast University, Suzhou, 215123, China; 3 Department of Chronic Disease Epidemiology, Yale School of Public Health, School of Medicine, Yale University, 60 College Street, New Haven, Connecticut, 06520–8034, United States of America; 4 Department of Obstetrics and Gynecology, Zhong Da Hospital, School of Medicine, Southeast University, Nanjing, 210009, China; National Institutes of Health, UNITED STATES

## Abstract

Human beings are inevitably exposed to ubiquitous phthalate esters (PAEs). Processed, packaged foods are popular nowadays, in which emulsifiers are frequently added as food additives. It is unclear how emulsifiers affect the bioavailability of ingested PAEs contaminants and their toxicities. The purposes of our study were to explore whether food emulsifier Glycerin Monostearate (GMS) could increase the internal exposure levels of six priority controlled PAEs and affect their reproductive toxicities when male rats are exposed to PAEs mixture (MIXPs). The male rats were exposed to MIXPs by gavage for thirty days in combination with or without given GMS. Phthalate monoesters (MPAEs), primary metabolites of PAEs, in rat urine were used as biomarkers to predict the internal exposure levels of the six PAEs, and their concentrations were determined using UPLC-MS. The reproductive toxicity was evaluated using serum testosterone levels test and histopathology of testes. Results showed that compared to PAEs exposure alone, the internal exposure levels of PAEs increased by 30%-49% in the presence of GMS. PAEs exposure led to the reduction of testosterone level by 23.4%-42.1% in the presence and absence of GMS, respectively, compared to the baseline. Testosterone levels in MIXPs+GMS and DEHP+GMS group were decreased by 9.1% and 13.6%, respectively, compared with MIXPs and DEHP group. Histopathology showed that injuries of testis (deciduous spermatids) were observed, and GMS exacerbated the injuries. The results indicated food emulsifiers chronically taken up might increase safety risks of food PAEs contaminants.

## Introduction

Phthalate esters (PAEs) are commonly used as plasticizer to increase the flexibility, pliability and elasticity of polyvinyl chloride (PVC) plastics, and also widely used in cosmetics, personal care products, food packaging, children’s toys and medical products [1–4]. Some PVC plastic products contain on average 30~45% PAEs by weight [4, 5]. Since PAEs are not chemically bound to the PVC, they can quite easily be released into environment from PVC products [6, 7]. With annual production of approximately 6.0 million tons worldwide, consumer products containing PAEs can cause human exposure directly through contact and use, indirectly through other products PAEs leaching into, or general environmental exposure. Humans are exposed to these compounds via ingestion, inhalation and dermal exposure through their whole lifetime, and ingestion is the primary exposure pathway [8–10]. PAEs can migrate into foods from plastic containers and/or environment, thereby indirectly entering human body. The total PAEs concentrations in fish from HongKong market ranged from 1.57 to 7.10 μg/g [11], and di(2-ethyhexyl) phthalate (DEHP) level was about 3 μg/g in pork from New York market [12]. Di(n-butyl) phthalate (DBP) and DEHP levels in cow milk packed in polyethlene containers reached 75 ng/mL and 195 ng/mL, respectively [13], and DEHP was about 3.6–101 ng/mL in light alcoholic drinks and soft drinks [14].

The total PAEs exposure of residents in Yangtze River Delta in China is estimated to be about 34–159 μg/kg/d, and DEHP exposure is about 16–116 μg/kg/d, with dietary intake accounting for over 90% of PAEs’ total intake [9, 15, 16]. The reference dose (RfD) or tolerable daily intake (TDI) of DEHP is 20 μg/kg/d assessed by US EPA [17], 37 μg/kg/d by EU Scientific Committee for Toxicity, Ecotoxicity and the Environment (CSTEE) [18], and 40–140 μg/kg/d by Japan health department [19]. Therefore, the TDI range of DEHP is 20–140 μg/kg/d, which nearly cover the PAEs and DEHP exposure level of the local residents in Yangtze River Delta.

PAEs are environmental endocrine disruptors (EEDs), with reproductive toxicity and metabolic toxicities [6, 20–22]. Therefore, phthalates dimethyl phthalate (DMP), diethyl phthalate (DEP), DBP, butyl benzyl phthalate (BBP), DEHP and di-*n*-octyl phthalate (DNOP) are classified by the United States Environmental Protection Agency (US EPA) as priority environmental pollutants [17, 23]. Epidemiological studies have reported that PAEs exposure might cause male reproductive toxicity [24, 25]. Wang et al. [26] reported that children in Yangtze River Delta of China were probably at a high risk of cumulative phthalate exposure. Furthermore, the toxicological effect of each component in PAEs mixture may have dose addition interaction [27] and potentially exacerbate their safety risks.

Since the primary exposure to PAEs is through oral intake, it is of great significance to study their digestion, absorption, metabolism and elimination. Some components of ingested food contaminants, i.e., bio-accessible contaminants, can be released from food matrix, through which the contaminants can be absorbed, and enter animal and human beings as the internal exposure dose. The factors such as food matrix, fat level and contaminants’ physicochemical property affect the contamination absorption.

Emulsifiers as food additives are widely used in food industry, and glycerin monostearate (GMS) accounts for about 50% of the total emulsifier consumption [28]. Emulsifiers are also used to improve the oral bioavailability of poorly absorbed medicines in pharmaceutical industry [29, 30], with concentrations typically lower than what used in food industry. In China, the reported intake levels of total emulsifiers are more than 254 mg/kg/d [31]. It has been hypothesized that if some factor(s) can increase the bioavailability of PAEs, the “tolerable exposure level” of PAEs may be intolerable, and consequently put health at risks. However, few studies have investigated whether emulsifiers can increase the internal exposure levels of PAEs, and enhance their toxicity, particularly in male reproductive system.

In this study, we aimed to investigate the effects of food emulsifier on PAEs’ internal exposure levels and male reproductive toxicities using the mixture of six priority controlled PAEs along with the most widely used food emulsifier GMS. Phthalate monoesters (MPAEs), the primary metabolites of PAEs in rat urine, were used as biomarkers to predict the internal exposure levels of the six PAEs, which were quantified using UPLC-MS. The toxicity was evaluated using serum testosterone level test and histopathology of testis. To our knowledge, this study is the first one to evaluate the impact of food emulsifiers on the internal exposure levels and toxicity of food contaminants.

## Materials and Methods

### Chemicals and reagents

**Fig 1:**
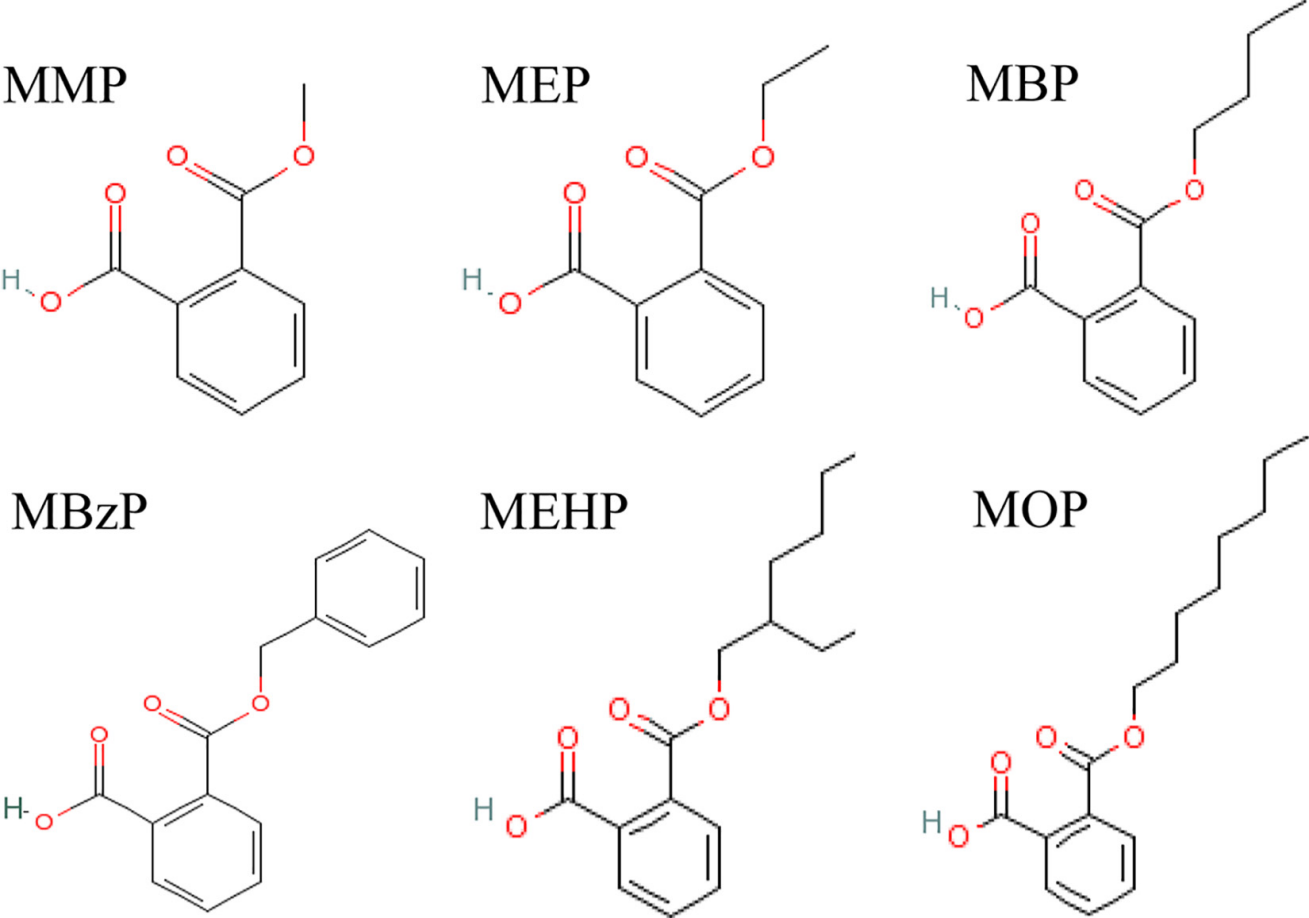
Chemical structures.

Monomethyl phthalate (MMP, CAS 4376-18-5), monoethyl phthalate (MEP, CAS 2306-33-4), monobutyl phthalate (MBP, CAS 131-70-4), monobenzyl phthalate (MBzP CAS 2528-16-7), mono-(2-ethylhexyl) phthalate (MEHP, CAS 4376-20-9) and mono-n-octyl phthalate (MOP, CAS 5393-19-1) were purchased from Accustandard Inc, USA. D4-MMP, D4-MEP, D4-MBP, D4-MBzP, D4-MEHP, D4-MOP were bought from CDN Inc., Canada. DMP, DEP, DBP, BBP, DEHP, DNOP, were supplied by Sinopharm Chemical Reagent CO. LTD, Shanghai, China. Metabolism cages were supplied by Fengshi laboratory animal equipment CO., LTD, Soochow, China. Auto-SPE machine (Preval SPE304+) was from Polytech Instrument. The chemical structures of MMP, MEP, MBP, MBzP, MEHP and MOP are shown in **Fig 1**.

### Animals

Male Sprague-Dawley rats (SPF grade, weighing 220–260 g) were purchased from Shanghai Jiesijie Experimental Animal CO. LTD (License Number: SCXK(HU)2013-0006) and were maintained in an environmentally controlled room (21 ± 2°C, 12 h dark-light cycle) with free access to the standard animal laboratory food for 7 days before experiments at SPF grade Animal Laboratory of Southeast University. Water was supplied *ad libitum* from glass bottles. Animal experiments were conducted following the “Principles of laboratory animal care” (NIH publication 86–23, revised 1986) and local regulations. Furthermore, all experiments were approved and supervised by the Animal Care and Use Committee and the Animal Ethics Committee at Southeast University (Approval Number: 2015-0123-006).

### Ultra-performance liquid chromatography-mass spectrometry analyses

An ultra-performance liquid chromatography (UPLC) method was established for the detection of phthalate monoesters (MMP, MEP, MBP, MBzP, MEHP and MOP), the primary metabolites of PAEs, in rat urine as biomarkers to predict the internal exposure levels of six PAEs. UPLC analysis was carried out using a Thermo Accela system. Sample separation was achieved on Betasil phenyl column (100 mm×2 mm, 3 μm) at a flow rate of 350 μL/min using a mobile phase of 0.1% acetic acid solution (phase A): methanol (phase B). A gradient elution started with 30% B over 4 min, followed by the first linear increase to 40% B over 4 min, the second linear increase to 50% B over 2 min, and the third linear increase to 70% B over 6 min with additional 4 min hold. At the end, a linear decrease to 30% B over 1 min with 1 min hold were applied. The column temperature was set to 30°C. The sample volume was 5 μL. The peak area ratios (A/A _I.S._) between the selected analytes and their corresponding internal standards (I.S.) were plotted against the concentrations of MPAEs. D4-MMP, D4-MEP, D4-MBP, D4-MBzP, D4-MEHP and D4-MOP, as the I.S., were utilized to quantitate MMP, MEP, MBP, MBzP, MEHP and MOP, respectively.

Mass spectrometry was carried out using a triple quadrupole tandem mass spectrometer (Thermo API4000) equipped with an electrospray interface (ESI) and MRM scan mode. Ions were created in the negative ion mode with the sprayer voltage -4.5 kV and the ion source temperature at 500°C. The pressure of collision gas, sheath gas, atomization gas and heat gas were 11.0, 22.0, 35.0, 20.0 psi, respectively.

Stock solutions of MPAEs (100 μg/mL) and isotopically labelled I.S. (D4-MPAEs, 5 μg/mL) in methanol were prepared. Standard solutions were a serial of consecutive dilutions of the stock solutions with methanol to the final concentrations in the range 0.01–10 μg/mL. Quality control solutions had three different concentrations of 50, 200 and 1,000 ng/mL of the solutions in methanol. All the stock solutions were stored at 4°C.

In order to avoiding the migration of PAEs from PVC to urine sample, all the materials and reagents have been kept away from plastic vessels or apparatus, and glass tubes were used in urine sample collection, store and test throughout this study.

The baseline levels of PAEs metabolites were determined using urine samples that were collected before PAEs exposure (namely, blank urine). The blank urine (900 μL) was spiked with 100 μL the quality control solutions with different concentrations, respectively, to make eight calibration standards at the final concentrations of 1, 10, 20, 50, 100, 200, 500 and 1,000 ng/mL. To prepare quality control samples at the final concentrations of 5, 20 and 100 ng/mL, the blank urine was spiked with the appropriate amount of quality control solutions, respectively. Fresh quality control samples were used to daily evaluate method parameters including extraction recovery, accuracy, precision and matrix effect. All the samples were stored at -20°C to maintain stability.

The matrix effect of each MPAEs was in the range of -11.33% to 5.95%, with relative standard deviation (RSD) <9%. Good recoveries (82%-119%) were observed over a linear range of 5–100 ng/mL, indicating that the method was fairly accurate. Thus, this method was valid for the determination of MPAEs in the urine samples.

### Sample preparation

The frozen urine samples (stored at -80°C freezers) were thawed under room temperature and subsequently filtrated through a 0.45 μm membrane filter. 1 mL urine sample in a 5 mL glass tube was mixed with 40 μL of D4-labelled I.S. mixture, followed by the addition of 250 μL of ammonium acetate buffer solution (pH = 6.5) and 20 μL of β-glucuronidase (140 U/mL, Sigma). The mixture was sealed and incubated in a water bath of 37°C for 90 min to hydrolyze the conjugates of glucuronic acid and phthalate metabolite to release MPAEs. After centrifugation at 4,000 rpm for 10 min, the supernatant was collected, and then diluted with 1 mL phosphate buffer solution (pH = 2.0) for further solid-phase extraction (SPE).

The dilution was passed through the pre-conditioned DVB SPE cartridges (with 1 mL acetonitrile and then 1 mL phosphate buffer solution) at a flow rate of 1 mL/min controlled by Auto-SPE vacuum pumping. 2 mL of formic acid (0.1 mol/L) and 1 mL of purified water was used to clean the cartridge. After cleaning, the pumping was kept on for another 1 min until the cartridge bed was completely dried. The target analytes retained on the cartridges were eluted with 1 mL acetonitrile. The eluent was evaporated to dry in 55°C water bath under nitrogen stream, and finally the dried substance was re-dissolved with 200 μL methanol for LC-MS/MS analysis.

### Internal exposure study

The mixture of PAEs (MIXPs) consisted of DMP, DEP, DBP, BBP, DEHP and DNOP at equipotent toxicity based on their RfD by weight (DMP: DEP: DBP: BBP: DEHP: DNOP = 10:0.8:0.1:0.2:0.02:3) [17]. 25 rats were divided randomly into five groups (n = 5). Rats in MIXPs group and MXIPs+GMS group received MIXPs suspension with 0.5% sodium carboxymethyl cellulose (CMC-Na) at the dose of 100 mg/kg/d with or without GMS (200 mg/kg/d) through intragastrical (ig) administration. The equivalent external exposure doses were 70.82 mg/kg/d for DMP, 5.67 mg/kg/d for DEP, 0.708 mg/kg/d for DBP, 1.42 mg/kg/d for BBP, 0.142 mg/kg/d for DEHP, and 21.25 mg/kg/d for DNOP, respectively. Rats in DEHP group and DEHP+GMS group received isometrical DEHP suspension with 0.5% CMC-Na at the dose of 100 mg/kg/d with or without GMS (200 mg/kg/d) by ig. The volume of the dose administered was 1.0 mL/100 g body weight. Rats in control group received isometrical 0.5% CMC-Na by ig. The treatments continued for 30 days. All rats were monitored each day for their health and mental status during the experimental period. Rats, except those in control group, were raised in metabolism cages to collect 24-hour urine on day 1 to day 7, and then on days 10, 15, 21, 27 and 30. The urine collected before exposure of rat to PAEs was collected to measure the background level of PAEs for self-control. No rat became severely ill during the experiments or died prior to the experimental endpoint. The urine samples were stored at -80°C before UPLC analysis. Urine creatinine was detected by Beckman Coulter DXC800 fully automatic biochemical analyzer.

The estimated daily intake of PAEs based on urine MPAEs concentration were regarded as internal exposure dose, which was calculated by formula (1) according to Chen *et al* [10] and David [32], and relative bioavailability (F_rel_) was estimated by formula (3) to compare the internal exposure dose of PAEs in the presence and absence of GMS.

$$\text{Internal exposure level}=\text{Daily intake}\;\left(\text{mg}/\text{kg}/\text{day}\right)=\text{UE}\;\left(\text{mg}/\text{g}\right)\times \text{CE}\;\left(\text{mg}/\text{kg}/\text{day}\right)÷{\text{F}}_{\text{UE}}\times \text{MWd}÷\text{MWm}$$ (1)

UE(mg/g)=MPAEs concentration(mg/L)in Urine÷Creatinine concentration(g/L)in Urine (2)

$${\text{F}}_{\text{rel}}=\text{Internal exposure level of MIXPs with GMS}÷\text{Internal exposure level of MIXPs}\times 100\%$$ (3)

where, UE is urinary excretion of MPAEs (mg/g creatinine) and calculated by the formula (2); We calculated CE (creatinine excretion) using the formula: CE = creatinine concentration× urine volume÷ body weight. CE is a constant and calculated to be 44 mg/kg/d for male rats in this study. F_UE_ is a fractional urinary excretion value of 0.69 for MEP [18] and MBP [33], 0.024 for MEHP [34], 0.73 for MBzP [33], and 0.043 for MOP [18]. DMP and DEP were assumed to have an equal excretion factor as DBP [10, 18]. MWd and MWm are molecular weight of PAEs and MPAEs, respectively.

### Toxicological effects on testis

At the beginning and end of the experiment, about 0.75 mL blood of each rat was collected from caudal vein by cutting off the tip of their tails, and centrifuged at 3000 rpm for 10 min at 4°C for serum preparation. Serum testosterone (T) level was tested using a commercial assay kit for T (Nanjing JianCheng Bioengineering Institute, Nanjing, China). All rats were fasted overnight (12 h) and euthanized using 4% fluothane in oxygen anesthesia in an airtight container. When the rats had no-blink reflex and no autonomous breath, or no heartbeat, the left testis of each rat was removed for fix in 10% buffered formalin, followed by embedding in paraffin. The 3–5 mm thick sliced sections were stained by Hematoxylin-Eosin. Morphological analyses were performed by an experienced pathologist blinded to the treatment information. The slides were examined by TS100 inverted microscope (Nikon, Japan), and photographed under ×200 magnification. The tissue sections were scored with respect to the degree of injury.

### Statistical analyses

All statistical analyses were performed using Statistical Product and Service software, version 17.0 (SPSS Inc., Chicago, IL, USA). Comparison between with and without GMS groups was performed using an independent-samples T test. Multiple comparisons were performed using one-way ANOVA; if the variances are homogenous, the least significant different t test is used, otherwise, a Tammany’s T2 test is used. Values are expressed as means and their corresponding standard deviations. Differences with *P* <0.05 were considered statistically significant.

## Results

### UPLC-MS analysis of MPAEs metabolites in urine

A good linear relationship was obtained for the six MPAEs with the concentrations ranging from 1.0 to 10,000 ng/mL. The linear regression parameters for the calibration curve of each phthalate monoesters are listed in Table 1. The correlation coefficients (R^2^) of all MPAEs were more than 0.99. The limit of detection (LOD) was 0.5 ng/mL for MMP, MEP, MBP and MBzP, and 0.3 ng/mL for MEHP and MOP. The limit of quantitation (LOQ) was 1.5 ng/mL for MMP, MEP, MBP and MBzP, and 1.0 ng/mL for MEHP and MOP, according to decuple signal-to-noise ratio. The coefficient of variation (CV) for each MPAE to determine inter- and intraday precision of 5, 20 and 100 ng/mL was <14.5%, indicating that the method was linear and reproducible.

**Table 1:** The urine-based calibration curve of each MPAE and their LOD and LOQ.

MPAEs	Calibration curve	R^2^	LOD(ng/ml)	LOQ(ng/ml)
MMP	Y = 0.0626X-0.0821	0.9984	0.5	1.5
MEP	Y = 0.0038X-0.0413	0.9934	0.5	1.5
MBP	Y = 0.0079X-0.0538	0.9997	0.5	1.5
MBzP	Y = 0.005X+0.0185	0.9996	0.5	1.5
MEHP	Y = 0.0609X+1.1775	0.9956	0.3	1.0
MOP	Y = 0.0284X+0.2017	0.9990	0.3	1.0

Note: Y represents concentration of MPAE in urine and X represents the peak area ratio of each MPAEs/MPAEs-D4

### GMS increases internal exposure level and relative bioavailability of PAEs

**Fig 2:**
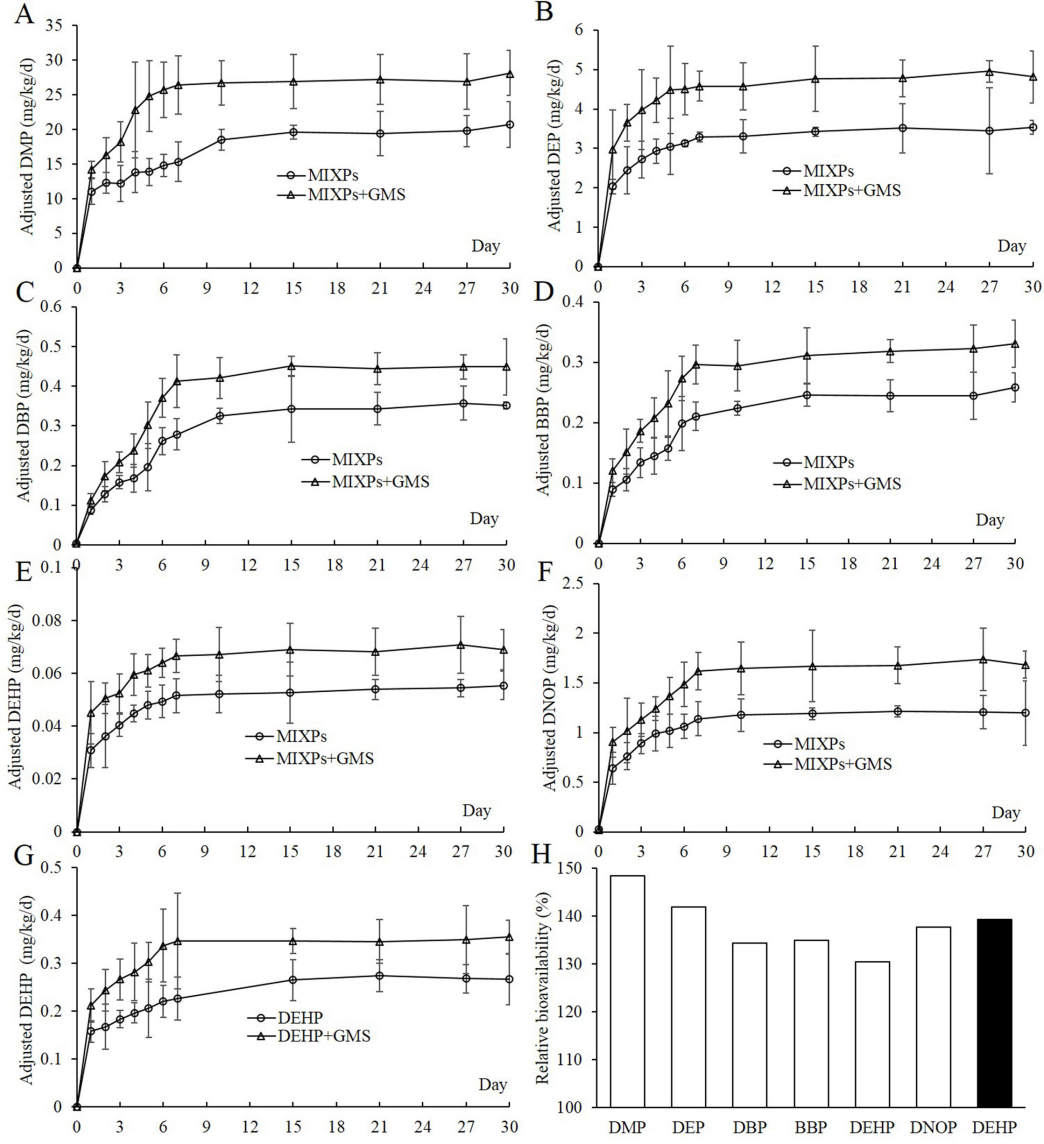
Internal exposure levels and relative bioavailability of PAEs; Mean internal exposure level profiles of DMP (A), DEP (B), DBP (C), BBP (D), DEHP (E) and DNOP (F) in the rats administered with MIXPs with or without GMS; Mean internal exposure level profile of DEHP (G) in the rats administered with DEHP alone at 100 mg/kg/d with or without GMS; (H), relative bioavailability of PAEs; (□), relative bioavailability of PAEs in rats administered with MIXPs at 100 mg/kg/d; (■), relative bioavailability of DEHP in rats administered with DEHP alone at 100 mg/kg/d.

Day 0 PAEs exposure levels in the rats were assigned as the background exposure for self-control. As shown in Table 2, the results showed that they were very low, even under the LOD. After the rats exposure to PAEs, the internal exposure levels of PAEs in the rats of MIXPs+GMS group were significantly higher than those of MIXPs group (*P* <0.05) (**Fig 2)**. The internal exposure levels of PAEs in rats continuously elevated in both MIXPs group and MIXPs+GMS group with a rapid increase in the first several days (**Fig 2A–2F**), and the F_rel_ of each PAEs ranged from 130% to 148% (**Fig 2H**). In addition, the internal exposure level of DEHP in the rats also continuously increased when administered with DEHP in the presence and absence of GMS (**Fig 2G**), and the DEHP F_rel_ in this group was 139%, obviously higher than DEHP F_rel_ (130%) in rats administered with MIXPs (**Fig 2H**), indicating the possibility of competitive absorption of PAEs.

**Table 2:** The background exposure level of the six priority controlled PAEs.

	DMP	DEP	DBP	BBP	DEHP	DNOP
MIXPs	2.52±1.52	1.51±0.40	3.34±1.63	ND	ND	27.57±14.92
MIXPs+GMS	1.61±1.28	1.30±0.49	3.47±1.77	ND	ND	19.68±7.97

Note: n = 5, unit: 10^−3^ mg/kg/d; ND, the background exposure level of BBP and DEHP were below the LOD of the detection method.

### Testis toxicity

**Fig 3:**
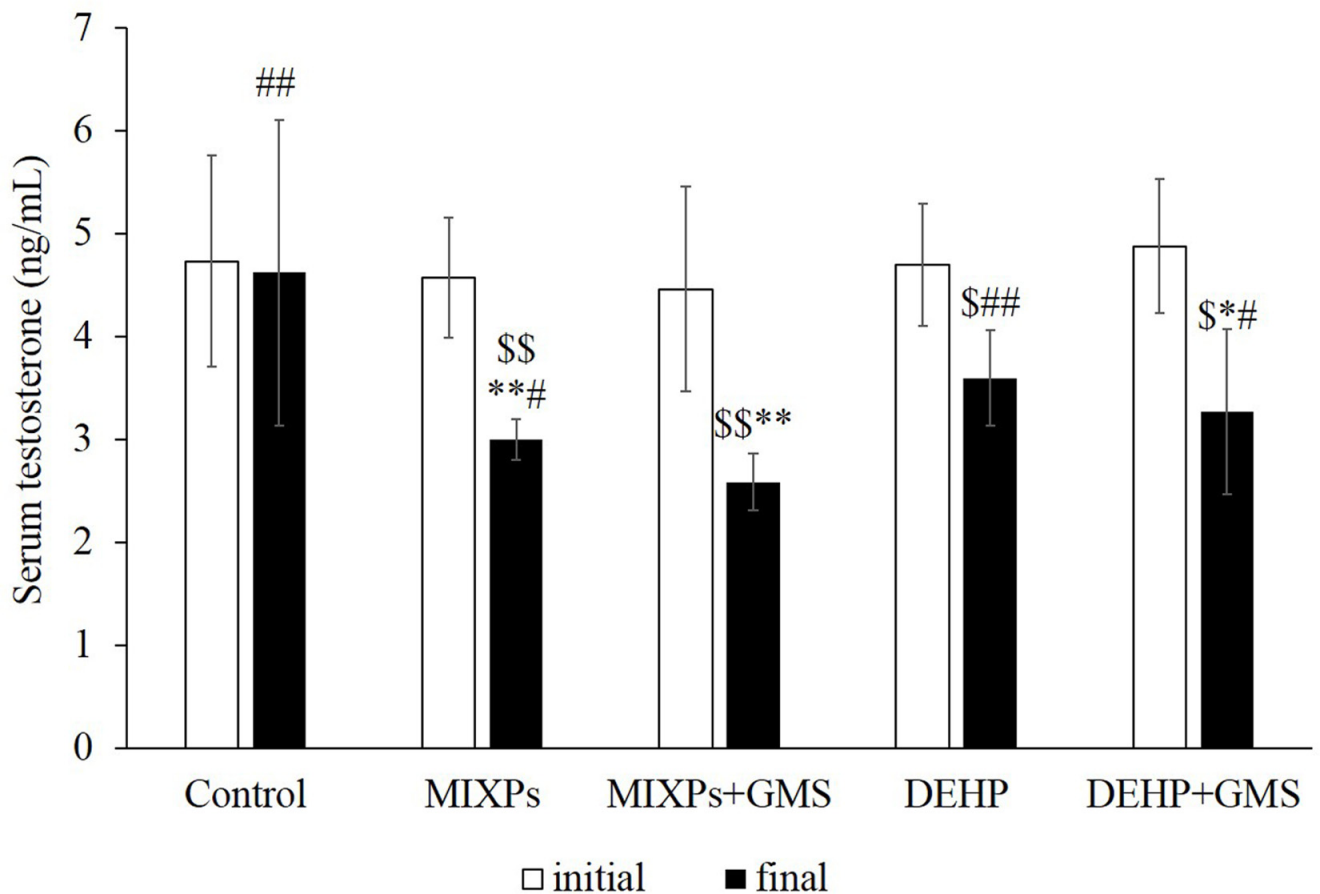
Initial and final serum testosterone levels in rats of each group. Comparing with self-initial, $<0.05, $ $<0.01; comparing with control, *<0.05, **<0.01; comparing with MIXPs+GMS, #<0.05, ##<0.01.

Serum T levels in experimental rats declined significantly (23.4%-42.1%) at the final stage of the experiment, compared to their self-initial stage and control rats (*P* <0.05). However, no different change of T level was observed over the period of experiment in control rats. As shown in **Fig 3**, T level in MIXPs+GMS group was reduced 9.1% comparing to MIXPs group (*P* <0.05). Similarly, T level in DEHP+GMS group was reduced 13.6% comparing to DEHP group. In addition, T levels in MIXPs group and MIXPs+GMS group were lower than DEHP group and DEHP+GMS group respectively, with notable differences between MIXPs and DEHP group (*P* <0.05). These results indicated that PAEs might pose reproductive toxicity and GMS could enhance their toxicities. Furthermore, there might have dose addition action among PAEs’ toxicological effects of each component.

**Fig 4:**
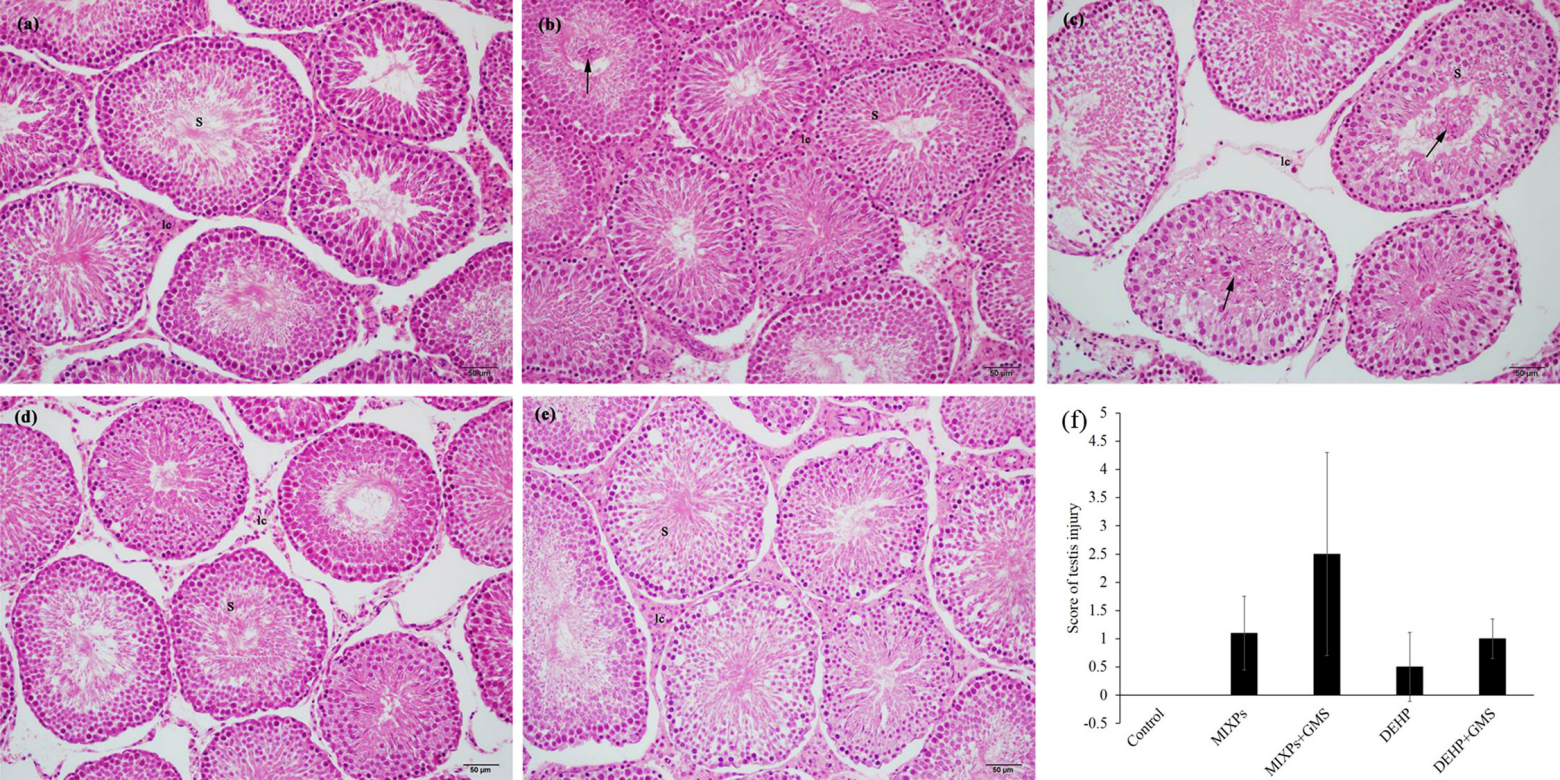
Histopathological images of rat testis in control group (a), MIXPs group (b), MIXPs+GMS group (c), DEHP group (d) and DEHP+GMS group (e), respectively. (f), Injury score. (S), seminiferous tubules; (Arrow), deciduous spermatids. Magnification ×200.

Pathological morphology of testis was examined since T is mainly generated by leydig cells in testis, a main target organ of PAEs. As shown in **Fig 4**, there were seminiferous tubules (S) with complete arrangement of germinal epithelium in control group with no injury (**Fig 4A**). Spermatids became sparse and plenty of deciduous spermatids (arrow) were observed in seminiferous tubules in MIXPs+GMS group (**Fig 4C**), while lots of sperm cells arranged orderly in the tubules and only a few deciduous spermatids were observed in MIXPs group (**Fig 4B**). No deciduous spermatids were observed in DEHP+GMS and DEHP group; nevertheless, the spermatids in DEHP+GMS group were sparser than those in DEHP group with disordered arrangement (**Fig 4D** and **4E**). The injury scores of MIXPs+GMS and DEHP+GMS group were higher than MIXPs and DEHP group, respectively (**Fig 4F**). The results of testicular pathological morphology were correlated with serum T levels, which supported the hypothesis that GMS enhanced PAEs’ reproductive toxicity. In addition, we also examined the morphological analyses of liver (**S1**). Similarly, the toxic effects were also observed in liver.

## Discussion

In human and animal body, PAEs are rapidly metabolized to their corresponding monoesters, and some of which can be further metabolized to the oxidation products of their lipophilic aliphatic side chain. For example, DMP is metabolized to MMP, while DEHP is metabolized to MEHP, which can be further metabolized to MEHHP, MEOHP and so on [5]. The simple monoester metabolites of PAEs are still the most common biomarkers to evaluate the exposure levels of their parent compounds [18, 33, 34]. There are some reports on the determination of MPAEs in urine, serum and milk by either HPLC or LC [35–37]. We have established HPLC method for analyzing MEHP and DEHP in plasma [38]. In this study, we developed a simple and rapid UPLC-MS method to determine the concentrations of MPAEs in rat urine and to evaluate the effects of food emulsifier GMS on PAEs internal exposure level. Theoretically, the UPLC-MS method has significant advantages in speed, resolution, and sensitivity of analysis, especially time saving and solvent consumption [39]. The UPLC-MS method developed in this study is validated with respect to the linearity range and the limit of detection. The retention time of MMP, MEP, MBP, MBzP, MEHP and MOP is 2.14, 7.54, 12.87 13.21, 18.58 and 19.20 min, respectively, with no interference from endogenous urine constituents at or near the retention time of those targeted analytes. The validated method has been applied to successfully determine the concentrations of MPAEs in urine sample in this study.

The usage of emulsifiers, such as GMS and polysorbate 20, in insoluble drugs is to improve their oral bioavailability by maintaining their stability and facilitating their release [29, 30, 40]. Emulsifiers are often used in food manufacture with higher concentrations, which may increase the oral bioavailability of food contaminants, such as PAEs. It has been reported that the oral bioavailability of DEHP in rats following single oral administration without emulsifier was 6.74% in the study of Chang-Liao *et al* [41] and 13.6% in the study of Pollack *et al* [42]. In our previous study, we found that polysorbate 80 could increase intestinal absorption of DEHP in rats, and the relative bioavailability of DEHP significantly increased by 1.9 folds [38]. In the present study, we found that food emulsifier GMS could increase the internal exposure level and oral bioavailability of six priority controlled PAEs, and the relative bioavailability of DMP, DEP, DBP, BBP, DEHP and DNOP increased by 1.48, 1.42, 1.34, 1.35, 1.30, and 1.39 folds, respectively.

Food emulsifiers are surfactants, which can reduce surface hydrophobicity, increase colonic permeability and make intestines sensitive to chemical aggression [43, 44]. P-glycoprotein (P-gp), locating in cytomembrane, plays an important role in protecting the body from harmful substances by limiting their entry into enterocytes and/or pump them out of enterocytes into the intestinal lumen [44, 45]. Zhu *et al* [30] found that polyoxyethylene (40) stearate (PS), a non-ionic surfactant, inhibited P-gp-mediated efflux by modulating substrate-stimulated P-gp ATPase activity. Similarly, another commonly used food emulsifier polysorbate 80, has inhibitive effect on P-gp [46]. Our previous study has demonstrated that polysorbate 80 could damage the structure and function of mitochondria in enterocytes, decrease mitochondrial respiration rate, and then inhibit the function of P-gp ATPase activity, leading to increased absorption of DEHP [38]. Barta *et al* [47] reported that GMS suppressed the activity and expression of P-gp in Caco-2 monolayer cells. Therefore, the potential mechanisms underlying the enhancement of PAEs’ internal exposure by GMS may include increased intestinal permeability and suppressed P-gp in enterocytes. An ongoing study is being conducted to understand the underlying mechanisms of the synergetic function of GMS in PAEs bioavailabilities.

PAEs are endocrine disruptors, and have reproductive and developmental toxicity [6, 48]. Liver and testis are main target organs of PAEs [49, 50]. Helal *et al* [48] reported that DEHP exposure at 1,000 mg/kg/d for 6 weeks could decrease testis weight and degenerate the germinal epithelium and leydig cells in rat testis. Diisobuty phthalate (DIBP), diisoheptyl phthalate (DIHP), diisononyl phthalate (DINP) and DEHP exposure induced the reduction of fetal testicular T production in SD and Wistar rat by inhibiting the expression of steroidogenesis genes such as steroidogenic acute regulatory protein (StAR) and Cyp11a [51]. DBP also decreased testicular T level in rat by down-regulating the expression of StAR, P450scc (P450 side-chain cleavage enzyme), 3β-HSD (3β-hydroxysteroid dehydrogenase), P450c17, which are key genes in steroidogenesis [52]. Phthalate esters have the similar structure of benzene ring and two adjacent aliphatic chains. They exert similar toxicological effects, such as disrupting the function of endocrine and male reproductive toxicity [17]. Howdeshell *et al* [27] found that exposure to the mixture of DEP, DBP, BBP, DEHP, and diisobuty phthalate (DiBP) inhibited fetal testicular T production in SD rat in a cumulative, dose-additive manner. Such effects are commonly reported for exposure to very high doses of PAEs (>500 mg/kg/d). In our study, with the exposure level of 100 mg/kg/d, deciduous spermatids were observed in MIXPs+GMS group, and spermatids became sparse in DEHP+GMS group, as well as T declined. These findings suggest that GMS exacerbated PAEs’ toxicity to testis. In addition, we also observed that GMS exacerbated PAEs’ toxicity to liver (**S1**). The exacerbated damage of testis and liver may result from increased internal exposure level of PAEs in the presence of GMS.

One of the pitfalls in most studies including ours, the experimental PAEs exposure levels are much higher than the everyday human exposure doses, which are usually low but chronic, continuous, and cumulative. Although the experimental doses cannot accurately reflect actual human exposure scenarios, widely used emulsifiers in foods may significantly increase the toxicities of PAEs by enhancing their bioavailabilities, thereby leading to human health risks. Several previous studies have reported that the elevated incidence of autoimmune diseases is associated with increased consumption food emulsifiers [44, 53, 54]. It will be very interesting to explore how food emulsifiers modify the toxicities of the chronic exposure to PAEs at low dose based on actual human exposure levels.

## Supporting Information

S1 Fig Histopathological images of rat liver. (DOCX)

## References

[1] Hauser R, Duty S, Godfrey-Bailey L, Calafat AM. Medications as a source of human exposure to phthalates. Environ Health Persp. 2004;112(6):751–3. 10.1289/Ehp.6804 15121520 PMC1241971

[2] Amberg-Muller JP, Hauri U, Schlegel U, Hohl C, Bruschweiler BJ. Migration of phthalates from soft PVC packaging into shower and bath gels and assessment of consumer risk. J Verbrauch Lebensm. 2010;5(3–4):429–42. 10.1007/s00003-010-0620-0

[3] Biedermann-Brem S, Biedermann M, Pfenninger S, Bauer M, Altkofer W, Rieger K, et al Plasticizers in PVC toys and childcare products: What succeeds the phthalates? Market survey 2007. Chromatographia. 2008;68(3–4):227–34. 10.1365/s10337-008-0672-9

[4] Bouma K, Schakel DJ. Migration of phthalates from PVC toys into saliva simulant by dynamic extraction. Food Addit Contam. 2002;19(6):602–10. 10.1080/02652030210125137 . 12042027

[5] Silva MJ, Samandar E, Preau JL, Needham LL, Calafat AM. Urinary oxidative metabolites of di(2-ethylhexyl) phthalate in humans. Toxicology. 2006;219(1–3):22–32. 10.1016/j.tox.2005.10.018 . 16332407

[6] Heudorf U, Mersch-Sundermann V, Angerer E. Phthalates: Toxicology and exposure. Int J Hyg Envir Heal. 2007;210(5):623–34. 10.1016/j.ijheh.2007.07.011 . 17889607

[7] Strac IV, Pusic M, Gajski G, Garaj-Vrhovac V. Presence of phthalate esters in intravenous solution evaluated using gas chromatography-mass spectrometry method. J Appl Toxicol. 2013;33(3):214–9. 10.1002/jat.1741 . 22034089

[8] Adibi JJ, Perera FP, Jedrychowski W, Camann DE, Barr D, Jacek R, et al Prenatal exposures to phthalates among women in New York City and Krakow, Poland. Environ Health Persp. 2003;111(14):1719–22. 10.1289/Ehp.6235 . 14594621 PMC1241713

[9] Chen L, Zhao Y, Li L, Chen B, Zhang Y. Exposure assessment of phthalates in non-occupational populations in China. Sci Total Environ. 2012;427–428(0):60–9. 10.1016/j.scitotenv.2012.03.090 . 22578696

[10] Chen ML, Chen JS, Tang CL, Mao IF. The internal exposure of Taiwanese to phthalate—An evidence of intensive use of plastic materials. Environ Int. 2008;34(1):79–85. 10.1016/j.envint.2007.07.004 . 17765308

[11] Cheng Z, Nie XP, Wang HS, Wong MH. Risk assessments of human exposure to bioaccessible phthalate esters through market fish consumption. Environ Int. 2013;57–58:75–80. 10.1016/j.envint.2013.04.005 . 23688402

[12] Schecter A, Lorber M, Guo Y, Wu Q, Yun SH, Kannan K, et al Phthalate concentrations and dietary exposure from food purchased in New York state. Environ Health Persp. 2013;121(4):473–9. 10.1289/ehp.1206367 . 23461894 PMC3620091

[13] Farajzadeh MA, Djozan D, Reza M, Mogaddam A, Norouzi J. Determination of phthalate esters in cow milk samples using dispersive liquid-liquid microextraction coupled with gas chromatography followed by flame ionization and mass spectrometric detection. J Sep Sci. 2012;35(5–6):742–9. 10.1002/jssc.201100853 . 22271644

[14] Russo MV, Notardonato I, Avino P, Cinelli G. Determination of phthalate esters at trace levels in light alcoholic drinks and soft drinks by XAD-2 adsorbent and gas chromatography coupled with ion trap-mass spectrometry detection. Anal Methods-Uk. 2014;6(17):7030–7. 10.1039/c4ay00926f

[15] Guo Y, Wu Q, Kannan K. Phthalate metabolites in urine from China, and implications for human exposures. Environ Int. 2011;37(5):893–8. 10.1016/j.envint.2011.03.005 . 21477864

[16] Yu YJ, Ye H, Yang Y, Zhao J. The bioaccessibility and exposure assessment of PAEs via oral media in Taihu Lake of south Jiangsu Province. Environ Chem. 2014;33(2):194–205. 10.7524/j.issn.0254-6108.2014.02.007

[17] US EPA. Phthalates summary 2007. URL: http://www.epa.gov/teach/chem_summ/phthalates_summary.pdf.

[18] Koch HM, Drexler H, Angerer J. An estimation of the daily intake of di(2-ethylhexyl)phthalate (DEHP) and other phthalates in the general population. Int J Hyg Envir Heal. 2003;206(2):77–83. 10.1078/1438-4639-00205 12708228

[19] Fujimaki K, Yoshinaga J, Watanabe C, Serizawa S, Shiraishi H, Mizumoto Y. Estimation of intake level of di (2-ethyhexyl) phthalate (DEHP) in Japanese pregnant women based on measurement of concentrations of three urinary metabolites. Nippon Eiseigaku Zasshi. 2006;61(3):340–7. 16768165 10.1265/jjh.61.340

[20] Ye T, Kang M, Huang Q, Fang C, Chen Y, Shen H, et al Exposure to DEHP and MEHP from hatching to adulthood causes reproductive dysfunction and endocrine disruption in marine medaka (Oryzias melastigma). Aquat Toxicol. 2014;146:115–26. 10.1016/j.aquatox.2013.10.025 . 24292025

[21] Tellez-Rojo MM, Cantoral A, Cantonwine DE, Schnaas L, Peterson K, Hu H, et al Prenatal urinary phthalate metabolites levels and neurodevelopment in children at two and three years of age. Sci Total Environ. 2013;461:386–90. 10.1016/j.scitotenv.2013.05.021 . 23747553 PMC3735862

[22] Bhatia H, Kumar A, Chapman JC, McLaughlin MJ. Long-term exposures to di-n-butyl phthalate inhibit body growth and impair gonad development in juvenile Murray rainbowfish (Melanotaenia fluviatilis). J Appl Toxicol. 2015;35(7):806–16. 10.1002/jat.3076 25348951

[23] Chen XP, Xu SS, Tan TF, Lee ST, Cheng SH, Lee FWF, et al Toxicity and estrogenic endocrine disrupting activity of phthalates and their mixtures. Int J Env Res Pub He. 2014;11(3):3156–68. 10.3390/ijerph110303156 . 24637910 PMC3987027

[24] Wang YX, Zeng Q, Sun Y, Yang P, Wang P, Li J, et al Semen phthalate metabolites, semen quality parameters and serum reproductive hormones: A cross-sectional study in China. Environ Pollut. 2016;211:173–82. 10.1016/j.envpol.2015.12.052 . 26766535

[25] Wang YX, Zeng Q, Sun Y, You L, Wang P, Li M, et al Phthalate exposure in association with serum hormone levels, sperm DNA damage and spermatozoa apoptosis: A cross-sectional study in China. Environ Res. 2015 10.1016/j.envres.2015.11.023 . 26654563

[26] Wang B, Wang H, Zhou W, Chen Y, Zhou Y, Jiang Q. Urinary excretion of phthalate metabolites in school children of China: implication for cumulative risk assessment of phthalate exposure. Environ Sci Technol. 2015;49(2):1120–9. 10.1021/es504455a . 25496010

[27] Howdeshell KL, Wilson VS, Furr J, Lambright CR, Rider CV, Blystone CR, et al A mixture of five phthalate esters inhibits fetal testicular testosterone production in the sprague-dawley rat in a cumulative, dose-additive manner. Toxicol Sci. 2008;105(1):153–65. 10.1093/toxsci/kfn077 . 18411233

[28] NHFPC. Food Additive Use Standards (GB2760-2014) National Health and Family Planning Commission of the People's Republic of China. Peking: Standards press of China; 2014.

[29] Alvi MM, Chatterjee P. A prospective analysis of co-processed non-ionic surfactants in enhancing permeability of a model hydrophilic drug. Aaps Pharmscitech. 2014;15(2):339–53. 10.1208/s12249-013-0065-8 . 24357111 PMC3969477

[30] Zhu SJ, Huang RQ, Hong MH, Jiang YY, Hu ZH, Liu C, et al Effects of polyoxyethylene (40) stearate on the activity of P-glycoprotein and cytochrome P450. Eur J Pharm Sci. 2009;37(5):573–80. 10.1016/j.ejps.2009.05.001 . 19442720

[31] Gao HT, Xu R, Cao WX, Yan YHM, Zhou X, Xu Q. Food safety risk related to food emulsifiers. Sci Technol Food Ind. 2015;36(23):280–4. 10.13386/j.issn1002-0306.2015.23.049

[32] David RM. Exposure to phthalate esters. Environ Health Persp. 2000;108(10):A440–A442. 10.1289/ehp.108-a440a PMC1240143 11097555

[33] Anderson WAC, Castle L, Scotter MJ, Massey RC, Springall C. A biomarker approach to measuring human dietary exposure to certain phthalate diesters. Food Addit Contam. 2001;18(12):1068–74. 10.1080/02652030110050113 . 11761117

[34] Koch HM, Drexler H, Angerer J. Response to the letter of R. M. David. Int J Hyg Envir Heal. 2004;207(1):77–8. 10.1078/1438-4639-00272 14762970

[35] Yoshimura M, Inoue K, Hanaoka T, Pan GW, Takahashi K, Yamano Y, et al Development of simultaneous determination method of phthalate monoester metabolites in urine by LC/MS/MS and its application to assessment of phthalate-ester exposure. Bunseki Kagaku. 2006;55(9):661–7. 10.2116/bunsekikagaku.55.661

[36] Mortensen GK, Main KM, Andersson AM, Leffers H, Skakkebwk NE. Determination of phthalate monoesters in human milk, consumer milk, and infant formula by tandem mass spectrometry (LC-MS-MS). Anal Bioanal Chem. 2005;382(4):1084–92. 10.1007/s00216-005-3218-0 . 15933851

[37] Calafat AM, Slakman AR, Silva MJ, Herbert AR, Needham LL. Automated solid phase extraction and quantitative analysis of human milk for 13 phthalate metabolites. J Chromatogr B. 2004;805(1):49–56. 10.1016/j.chromb.2004.02.006 15113539

[38] Lu Y, Wang YY, Yang N, Zhang D, Zhang FY, Gao HT, et al Food emulsifier polysorbate 80 increases intestinal absorption of di-(2-ethylhexyl) phthalate in rats. Toxicol Sci. 2014;139(2):317–27. 10.1093/toxsci/kfu055 . 24675089

[39] Wu T, Wang C, Wang X, Xiao HQ, Ma Q, Zhang Q. Comparison of UPLC and HPLC for Analysis of 12 Phthalates. Chromatographia. 2008;68(9–10):803–6. 10.1365/s10337-008-0788-y

[40] Xia D, Cui F, Gan Y, Mu H, Yang M. Design of lipid matrix particles for fenofibrate: effect of polymorphism of glycerol monostearate on drug incorporation and release. J Pharm Sci-US. 2014;103(2):697–705. 10.1002/jps.23830 . 24375427

[41] Chang-Liao WL, Hou ML, Chang LW, Lee CJ, Tsai YM, Lin LC, et al Determination and pharmacokinetics of Di-(2-ethylhexyl) phthalate in rats by ultra performance liquid chromatography with tandem mass spectrometry. Molecules. 2013;18(9):11452–66. 10.3390/molecules180911452 . 24043141 PMC6269943

[42] Pollack GM, Li RCK, Ermer JC, Shen DD. Effects of Route of Administration and Repetitive Dosing on the Disposition Kinetics of Di(2-Ethylhexyl) Phthalate and Its Mono-De-Esterified Metabolite in Rats. Toxicol Appl Pharm. 1985;79(2):246–56. 10.1016/0041-008x(85)90346-1 4002226

[43] Lugea A, Salas A, Casalot J, Guarner F, Malagelada JR. Surface hydrophobicity of the rat colonic mucosa is a defensive barrier against macromolecules and toxins. Gut. 2000;46(4):515–21. 10.1136/Gut.46.4.515 10716681 PMC1727902

[44] Csáki KF. Synthetic surfactant food additives can cause intestinal barrier dysfunction. Med Hypotheses. 2011;76(5):676–81. 10.1016/j.mehy.2011.01.030 . 21300443

[45] Hennessy M, Spiers JP. A primer on the mechanics of P-glycoprotein the multidrug transporter. Pharmacol Res. 2007;55(1):1–15. 10.1016/j.phrs.2006.10.007 . 17095241

[46] Zhang HJ, Yao M, Morrison RA, Chong SH. Commonly used surfactant, tween 80, improves absorption of P-glycoprotein substrate, digoxin, in rats. Arch Pharm Res. 2003;26(9):768–72. 10.1007/Bf02976689 14560928

[47] Barta CA, Sachs-Barrable K, Feng F, Wasan KM. Effects of monoglycerides on P-glycoprotein: modulation of the activity and expression in Caco-2 cell monolayers. Mol Pharm. 2008;5(5):863–75. 10.1021/mp800050q . 18651749

[48] Helal MAM. Celery oil modulates DEHP-induced reproductive toxicity in male rats. Reprod Biol. 2014;14(3):182–9. 10.1016/j.repbio.2014.04.002 . 25152515

[49] Rusyn I, Peters JM, Cunningham ML. Modes of action and species-specific effects of di-(2-ethylhexyl)phthalate in the liver. Crit Rev Toxicol. 2006;36(5):459–79. 10.1080/10408440600779065 . 16954067 PMC2614359

[50] Moffit JS, Bryant BH, Hall SJ, Boekelheide K. Dose-dependent effects of sertoli cell toxicants 2,5-hexanedione, carbendazim, and mono-(2-ethylhexyl) phthalate in adult rat testis. Toxicol Pathol. 2007;35(5):719–27. 10.1080/01926230701481931 . 17763286

[51] Erkekoglu P, Zeybek ND, Giray B, Asan E, Arnaud J, Hincal F. Reproductive toxicity of di(2-ethylhexyl) phthalate in selenium-supplemented and selenium-deficient rats. Drug Chem Toxicol. 2011;34(4):379–89. 10.3109/01480545.2010.547499 . 21714771

[52] Barlow NJ, Phillips SL, Wallace DG, Sar M, Gaido KW, Foster PMD. Quantitative changes in gene expression in fetal rat testes following exposure to Di(n-butyl) phthalate. Toxicol Sci. 2003;73(2):431–41. 10.1093/toxsci/kfg087 . 12700402

[53] Roberts CL, Rushworth SL, Richman E, Rhodes JM. Hypothesis: Increased consumption of emulsifiers as an explanation for the rising incidence of Crohn's disease. J Crohns Colitis. 2013;7(4):338–41. 10.1016/j.crohns.2013.01.004 . 23360575

[54] Lerner A, Matthias T. Changes in intestinal tight junction permeability associated with industrial food additives explain the rising incidence of autoimmune disease. Autoimmun Rev. 2015;14(6):479–89. 10.1016/j.autrev.2015.01.009 . 25676324

