# Supplementary material for: Food Emulsifier Glycerin Monostearate Increases Internal Exposure Levels of Six Priority Controlled Phthalate Esters and Exacerbates Their Male Reproductive Toxicities in Rats
Source: PLoS One. 2016 Aug 30;11(8):e0161253. doi: 10.1371/journal.pone.0161253 (PMC5004918; doi:10.1371/journal.pone.0161253)
Supplement: S1 Fig — (DOCX) [file pone.0161253.s001.docx]

**S1. Histopathological images of rat liver**

The corresponding location of the left livers were removed, fixed in 10% buffered formalin, embedded in paraffin and sliced in 3-5 mm thick sections. They were stained by Hematoxylin-Eosin (H E). Morphological analyses were performed by an experienced pathologist blinded to the source of the tissues.

The structure of rat’s hepatic lobule, hepatic cord (hc) and hepatic sinusoid (hs) were distinguishable in control group with normal arrangement (**S1 a**). Fat cavitation (arrow) and hepatocyte ballooning (arrow head) respectively indicated fatty liver and hepatic edema. They were observed in rats of DEHP+GMS group (**S1 e**) and MIXPs group (**S1 b**). More large fat cavitations were observed in MIXPs+GMS group (**S1 c**). No injury was found in DEHP group (**S1 d**). Their injury scores were shown in **S1 f**. The injury scores of MIXPs+GMS and DEHP+GMS group were respectively higher than MIXPs and DEHP group (**S1 f**). This indicated that GMS also reinforced PAEs’ toxicity to liver in rats regardless of being administered with MIXPs or DEHP alone.

**
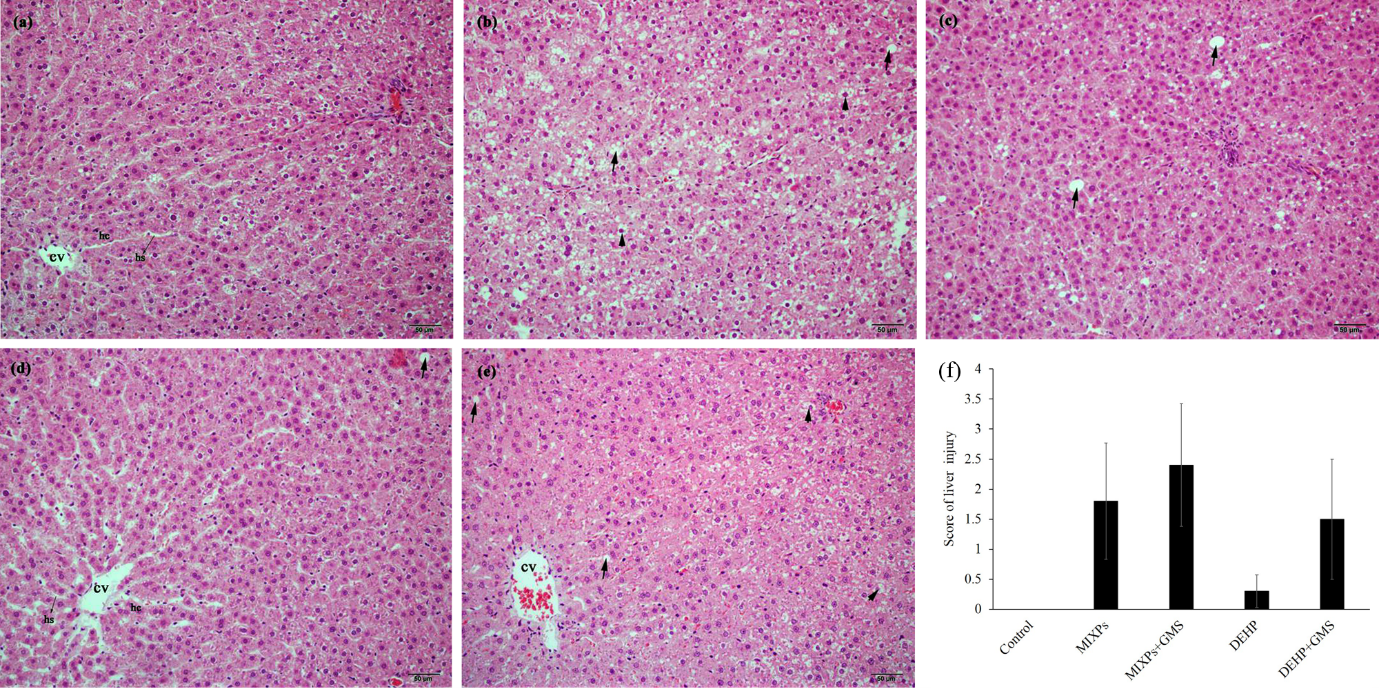
**

**S1.** Histopathological images of rat liver in control group (a), MIXPs group (b), MIXPs+GMS group (c), DEHP group (d) and DEHP+GMS group, respectively. (f), Injury score. (CV), central vein; (hc), hepatic cord; (hs), hepatic sinusoid; (Arrow), fat cavitation; (Arrow head), ballooning degeneration. Magnification ×200. The scoring criteria and the original data of score is available on the online database: https://yunpan.cn/cBpuH3g9bWRvE, password: 9000.
